# Evidence of more ion channels inhibited by celecoxib: K_V_1.3 and L-type Ca^2+^ channels

**DOI:** 10.1186/s13104-015-1023-1

**Published:** 2015-03-01

**Authors:** Roman V Frolov, Satpal Singh

**Affiliations:** Department of Physical Sciences, Division of Biophysics, University of Oulu, P.O. Box 3000, Oulun Yliopisto, 90014 Finland; Department of Pharmacology and Toxicology, State University of New York at Buffalo, Buffalo, NY 14214 USA

**Keywords:** Celecoxib, Ion channels, K_V_1.3, L-type Ca^2+^ channels

## Abstract

**Background:**

Celecoxib, a selective inhibitor of cyclooxygenase-2, can directly modulate many voltage-activated potassium, sodium and calcium channels and alter functioning of excitable cells. The inhibitory and facilitating effects of celecoxib on ion channels occur at low micromolar concentrations, bordering on therapeutic concentrations achievable in the clinical setting. The experiments described here were performed with the goals (1) to increase the range of ion channels tested, and (2) to examine possible differences in celecoxib’s effects on channels from different species.

**Findings:**

The channels examined in this study using patch-clamp and intracellular recording methods were human K_V_1.3 channels expressed in CHO cells, L-type Ca^2+^ channels (LTCC) from guinea pig cardiomyocytes, and LTCCs from *Drosophila* larval body-wall muscles. Celecoxib inhibited K_V_1.3 currents with IC_50_ of 5.0 μM at the end of 200 ms pulses to +20 mV. Celecoxib inhibited peak currents through guinea pig and *Drosophila* LTCCs with IC_50_s of 10.6 and 76.0 μM, respectively.

**Conclusions:**

As blockade of K_V_1.3 channels is associated with suppression of inflammatory immune reactions, the finding that celecoxib can inhibit these channels raises a question of possible contribution of K_V_1.3 inhibition to the anti-inflammatory effects of celecoxib. On the other hand, the Ca^2+^ channel results are consistent with previous observations indicating that, in contrast to K^+^ channels, strength of celecoxib effects on LTCCs strongly varies from species to species.

## Findings

Numerous studies have shown that celecoxib, a highly popular inhibitor of cyclooxygenase-2 (COX-2), can directly interact with many molecular targets unrelated to COX-2, including ion channels. Celecoxib inhibits Na_V_1.5 [1] (and other voltage-activated Na^+^ channels [2,3]), L-type Ca^2+^ channels (LTCC) [4,5], T-type Ca^2+^ channels [6], K_V_1.5 [7], K_V_2.1 [7,8] (and also *Drosophila* K_V_2 [9,10]), K_V_4.3 [1,7], K_V_7.1 [1,7,11], K_V_11.1 [1], while stimulating K_V_7.2-5 channels [5,11,12] at low micromolar concentrations independently of cyclooxygenase inhibition. Substantial and sometimes dramatic changes in cellular and tissue physiology following modulation of ion channel function by celecoxib have been also described (reviewed in [13]).

The experiments described in this report were performed with the aims (1) to increase the number of ion channels tested with celecoxib, and (2) to examine possible differences in celecoxib’s effects on homologous ion channels from different experimental systems. These goals address the need to conduct a comprehensive research of celecoxib actions on ion channels, including drug testing in different experimental systems, as differences between human and animal cardiac excitability usually do not allow reliable conclusions on the basis of a few animal studies.

### Experimental procedures

#### Patch-clamp

Experiments involving measurements of K_V_1.3 channels stably expressed in CHO cells and L-type Ca^2+^ channels in guinea pig ventricular myocytes were performed by Dr. David Rampe (Aventis Pharmaceuticals). Experimental procedures and protocols were approved by the Sanofi-Aventis Institutional Animal Care and Use Committee (Bridgewater, NJ) and conform to the Guide for the Care and Use of Laboratory Animals published by the National Institutes of Health. Single ventricular myocytes were isolated from guinea pigs as described previously [14]. Briefly, currents were recorded using an Axopatch 200B amplifier (Axon Instruments, CA, USA). Electrodes for whole-cell recordings (1–4 MΩ resistance) were made from TW150F glass capillary tubes (WPI, Sarasota, FL). For K_V_1.3 channel recordings, the electrode solution contained (in mM): potassium aspartate (120), KCl (20), disodium adenosine triphosphate (4), HEPES (5), and MgCl_2_ (1), pH 7.2 with KOH. The external solution contained: NaCl (130), KCl (5), sodium acetate (2.8), MgCl_2_ (1), HEPES (10), glucose (10), CaCl_2_ (1), pH 7.4 with NaOH. For Ca^2+^ channel recordings, the electrode solution contained: cesium methanesulfonate (130), tetraethylammonium chloride (20), MgCl_2_ (1), EGTA (10), HEPES (10), Tris-ATP (4), Tris-GTP (0.3), phosphocreatine (14), 50 U/ml creatine phosphokinase, pH 7.2 with CsOH. The bath solution contained: NaCl (137), CsCl (5.4), CaCl_2_ (1.8), MgCl_2_ (1), HEPES (10), glucose (10), pH 7.4 with NaOH.

#### Intracellular recordings

L-type currents were recorded using the two-microelectrode voltage-clamp technique from larval ventral longitudinal muscle 12 as described previously [15]. Electrodes were pulled from thin-walled borosilicate glass capillaries (World Precision Instruments, Sarasota, FL) using a Sutter Instruments puller, model P-97. Electrodes for potential measurements were filled with 2.5 M KCl, while current passing electrodes were filled with a 3:1 mixture of 2.5 M KCl and 2 M potassium citrate. Resistance of electrodes was between 8 and 14 MΩ. Holding potential of–40 mV was used to inactivate T-type Ca^2+^ channels [16]. Recording saline contained (in mM): NaCl (77.5), KCl (5.0), MgCl_2_ (4), NaHCO_3_ (2.5), trehalose (5.0), sucrose (115.0), HEPES (5.0), tetraethylammonium (TEA) (20.0), 4-aminopyridine (4-AP) (1.0), quinidine (0.1), and BaCl_2_ (10.0), pH 7.1 with NaOH. Voltage-activated potassium currents were blocked by TEA, quinidine, and 4-AP. Barium was used as charge carrier to reduce muscle contractions, to further inhibit K^+^ channels, and to eliminate Ca^2+^-dependent inactivation of LTCCs. To reduce the run-down error, only a single run of the experimental protocol was used to record from each muscle fiber.

All experiments were performed at room temperature. All values are means ± s.e.m; (*n*) indicates the number of experiments. Statistical analysis was performed by unpaired *t*-test; P < 0.05 was considered significant.

### Effects of celecoxib on K_V_1.3 channels

Delayed rectifier K_V_1.3 channels are found in T and B lymphocytes where they regulate membrane potential and calcium signaling [17,18]. K_V_1.3 channels are physically coupled to the T-cell receptor signaling complex through a series of adaptor proteins and play an essential role in T-cell proliferation and activation [19-23]. Blockade of K_V_1.3 channels in effector-memory T-cells suppresses calcium signaling, cytokine production and cell proliferation [20,21,23]. *In vivo*, K_V_1.3 blockers paralyze effector-memory T-cells at sites of inflammation and prevent their reactivation in inflamed tissues. Modulation of K_V_1.3 channels may be one of many mechanisms that contribute to apoptosis [24,25].

**Figure 1:**
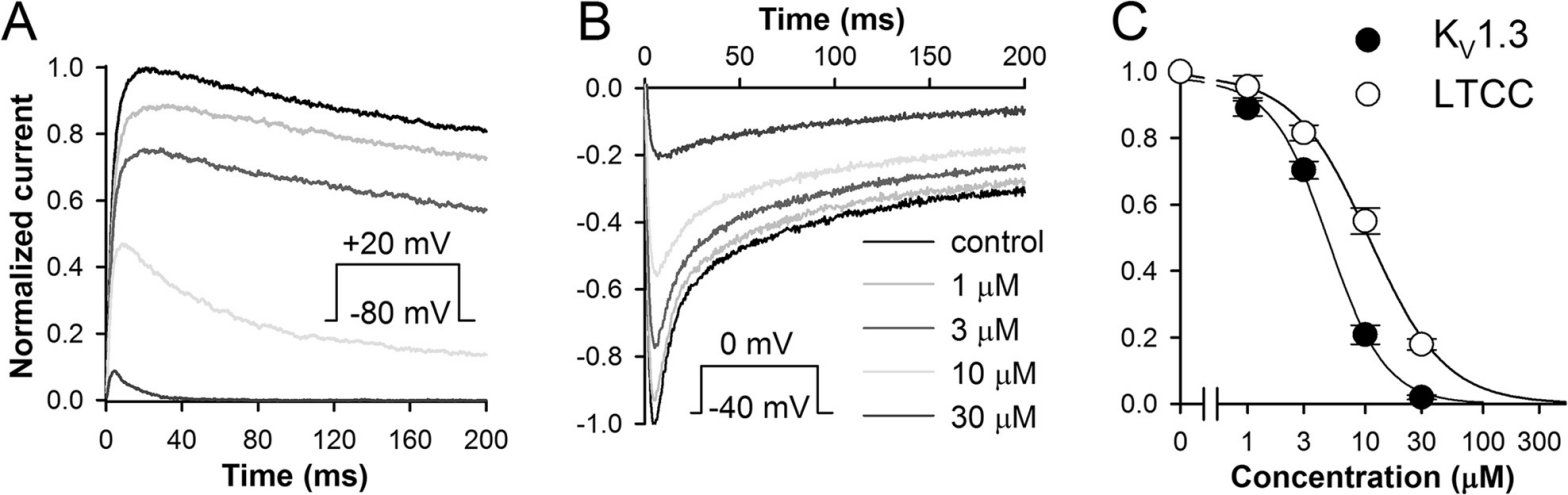
**Inhibition of K**_**V**_ **1.3 channels expressed in CHO cells and LTCCs in guinea pig ventricular myocytes by celecoxib. (A)** Representative examples of K_V_1.3 currents evoked by 200 ms pulses to +20 mV from a holding potential (HP) of -80 mV in control and after application of different concentrations of celecoxib (see legend in the panel **B**); responses of the same cell are shown. **(B)** Inhibition of LTCCs in guinea pig ventricular myocytes. Currents were evoked from a HP of -40 mV with a voltage pulse to 0 mV; representative examples of current traces from the same cell are shown; 1.8 mM Ca^2+^ was used as a charge carrier. **(C)** Concentration-dependences for inhibition by celecoxib of K_V_1.3 current at the end of 200 ms pulse (*n*=6) as well as peak LTCC current *(n*=5); data points were fitted with Hill equation.

In this study, effects of celecoxib on human K_V_1.3 channels expressed in CHO cells were examined using patch-clamp method. Figure 1A,C shows that celecoxib can inhibit whole-cell K_V_1.3 currents with an IC_50_ of 5.0 μM (for the currents at the end of 200 ms pulses). Note that celecoxib accelerated inactivation of the current, which indicates that the drug effect may be stronger during longer depolarizations.

Since blockade of K_V_1.3 channels is associated with suppression of inflammatory immune and autoimmune reactions, these findings raise a question about possible contribution of K_V_1.3 inhibition into the anti-inflammatory action of celecoxib, making a strong case for further research in this direction.

### Effects of celecoxib on LTCCs in Drosophila and guinea pig

We assessed effects of celecoxib on L-type Ca^2+^ channels in two model systems, guinea pig ventricular myocytes and *Drosophila* larval body-wall muscles. The rationale behind this test was two-fold. Firstly, although two reports have previously addressed inhibition of L-type Ca^2+^ channels by celecoxib in undifferentiated pheochromocytoma (PC12) cells [4] and in A7r5 rat aortic smooth muscle cells [5], no study has been undertaken in proper cardiac myocytes. Secondly, unlike the highly consistent effects of celecoxib on several other ion channels [5,7,10,11,26], the potency of L-type Ca^2+^ channel inhibition differed between the two published reports by nearly 20-fold (IC_50_s of 0.45 and 8.3 μM) [4,5], which is a significant difference in the clinical context.

In the human heart, L-type calcium channels (Ca_v_1) provide an influx of Ca^2+^ and mediate the excitation–contraction coupling. Mutations in Ca_v_1.2 can cause the Timothy syndrome which features congenital heart disease and may culminate in the sudden death from arrhythmia [27]. In this work, effects of celecoxib on cardiac L-type Ca^2+^ channels were tested in isolated guinea pig ventricular myocytes using patch-clamp method. Celecoxib inhibited whole-cell peak L-type Ca^2+^ currents with an IC_50_ of 10.6 μM (Figure 1B,C), a value very close to that obtained in A7r5 cells [5].

*Drosophila* larval body-wall muscles are mainly employed for studies of ion channels and synaptic transmission in the neuromuscular junction [28,29]. Several ionic currents are found in these muscles: T-type and L-type Ca^2+^ currents; two calcium-activated K^+^ currents; and three voltage-activated K^+^ currents including *I*_A_ (Shaker), *I*_KS_ (Shab), and *I*_KF_ (molecular basis unknown) [30]. We recorded Ba^2+^ currents through LTCCs in *Drosophila* larval body-wall muscles using the two-electrode voltage-clamp method.

**Figure 2:**
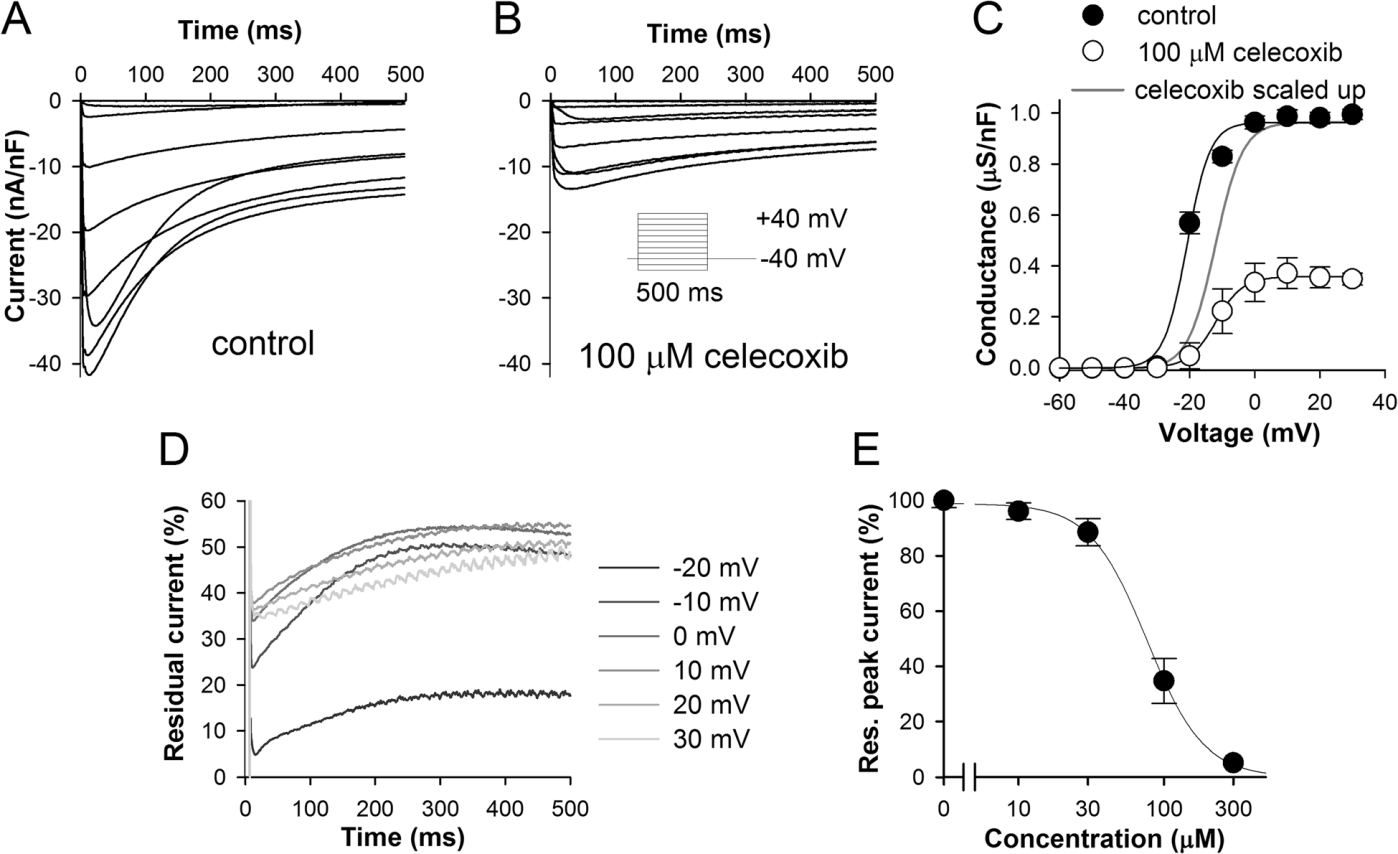
**Inhibition of L-type Ca** ^**2+**^ **channels in** ***Drosophila*** **larval body-wall muscles by celecoxibc. (A)** Average LTCC currents in control evoked by 500 ms pulses to 0 mV from a HP of -40 mV using 10 mM Ba^2+^ as charge carrier. **(B)** Average LTCC current in the presence of 100 μM celecoxib. **(C)** Effects of 100 μM celecoxib on voltage-dependence of LTCC activation; the dark gray scaled up trace illustrates the depolarizing shift of activation curve in the presence of 100 μM celecoxib. **(D)** Voltage-and time-dependence of inhibition; traces were obtained by dividing the average current traces in the presence of 100 μM celecoxib (panel **B**) by the average traces in control (panel **A**); first 5 ms were skipped. **(E)** Concentration-dependence for peak LTCC current inhibition by celecoxib at 0 mV; number of experiments for each concentration varied from 3 to 7.

Figure 2A shows average LTCC currents in control experiments evoked from a holding potential of-40 mV by voltage steps from-60 to 40 mV in 10 mV increments. Celecoxib inhibited *Drosophila* LTCCs in concentration-and voltage-dependent manner (Figure 2B-E). Figure 2C demonstrates the effect of celecoxib on the half-activation potential (V^a^_½_) of LTCCs. V^a^_½_ was -20.3 ± 0.5 mV (*n*=21) in control but -10.3 ± 1.3 mV (*n*=6) in the presence of 100 μM celecoxib (P < 0.001). Moreover, it follows from Figure 2D that celecoxib disproportionally reduced peak LTCC currents and that the inhibition increased during first several milliseconds after the onset of depolarization. These results are not consistent with the open-channel block, which is usually associated with increase in inactivation and a hyperpolarizing shift in the V^a^_½_ value, nor with closed-channel block, which is usually strongest at the beginning of a depolarizing pulse and gets gradually relieved during depolarization [13]. In contrast to the guinea pig, celecoxib inhibited *Drosophila* LTCCs with relatively low affinity (an IC_50_ of 76.0 μM, Figure 2E).

These results imply a substantial variability in celecoxib’s action on Ca^2+^ channels in different experimental systems. It is not known whether such variability stems from differences in the expressed channel isoforms (alpha-1 subunit of *Drosophila* voltage-dependent Ca^2+^ channel type D expressed in larval body-wall muscles [31] and alpha-1C subunit of guinea pig cardiac Ca^2+^ channel [32] are only 62% identical when the proteins are aligned using BLAST), or from dissimilar mechanisms of drug action. However, it seems to be unlikely that differences in recording techniques *per se* could contribute to the observed difference in celecoxib potency. For instance, celecoxib inhibits K_V_2 channels in the same *Drosophila* larval body-wall muscle preparation and in mammalian cell lines with very similar IC_50_s [13]. Moreover, effects of celecoxib on LTCCs do not depend on a particular charge carrier [5] even though using barium abolishes rapid calcium-dependent inactivation of LTCCs [15].

Although celecoxib potency differences reported here are likely to originate from molecular dissimilarities between the guinea pig and *Drosophila* L-type Ca^2+^ channels, this can hardly be the case for celecoxib effects on LTCCs in rat PC12 and A7r5 cells [4,5]. The LTCC IC_50_ discrepancies between rat PC12 and A7r5 cells imply that the open channel block is an unlikely mechanism of drug action, because the ability of a drug molecule to bind to its site in the channel pore and mechanically block the ion flow should not be affected that drastically by presumably dissimilar channel milieus in different cell types. Rather, these observations favor the gating modification mechanism previously described for celecoxib’s interaction with some other channels [26]. Biophysical properties of calcium channels are regulated by factors such as auxiliary subunits, which can be differentially expressed in different cell types [33] and thus the extent of channel modulation by celecoxib can be affected by tissue-specific modification of channel kinetics by these or other factors.
